# Agneta Montgomery—A Role Model

**DOI:** 10.3389/jaws.2024.12842

**Published:** 2024-05-10

**Authors:** Nadia A. Henriksen, Marc Miserez

**Affiliations:** ^1^ Department of Gastrointestinal and Liver Diseases, Herlev Hospital, Herlev, Denmark; ^2^ Department of Abdominal Surgery, UZ Leuven, KULeuven, Brussels, Belgium

**Keywords:** diversity, women in science, women in surgery, EHS, parastomal hernia repair

## Agneta Montgomery and the EHS

Agneta, as part of the #herniafamily, is synonymous with the EHS. She was active within the EHS from the early 1990’s when it was still a very small society run by a hernia elite of European men, collaborating strongly with American hernia surgeons. For many years (2013–2020) she was a board member (of Education, Journal Hernia, President-elect) taking over the Presidency of the EHS from Professor Marc Miserez (her Co-Editor at Hernia) in 2019.

We know her to be an excellent surgeon, scientist, President, and friend. She demonstrated a unique blend of authentic integrity, drive, honesty, and the capacity to bring surgeons (sometimes fractious) together (which was at times, necessary). She charmingly inspired and admired her colleagues so enthusiastically that it brought out the best in them, and indeed in us. Her work on guideline committees, the Swedish registry, and co-organisation of the fantastic Copenhagen 2021 joint EHS/AHS Congress need mentioning. The EHS owes Agneta everlasting respect and gratitude for an amazing career, and devoted service to the EHS. A role model for so many, an inspiration to more and a friend and great surgeon to all. We wish her all the very best in her retirement.

On behalf of the EHS board,

Maarten Simons, President.

Andrew deBeaux.

## Agneta Montgomery—A Role Model

Nadia A. Henriksen, Marc Miserez.

Agneta Montgomery was born in 1955 in Sweden. Agneta grew up in the countryside with her twin brother and older sister. Both of her parents were teachers at the local school, although her father’s dream was always to become a surgeon. When she was growing up, Agneta designed and sewn almost all of her clothes. Probably this was already an early sign that sewing would become an important part of her later career. Agneta began her medical studies at the University of Lund in 1974, got her degree as an MD in 1982, and became a specialized surgeon 6 years later. Since then, she has been working as a consultant surgeon at the Department of Surgery, Malmö hospital, Sweden.

Agneta started clinical research early in her career and defended her PhD thesis in 1995 entitled “*Intramucosal pH of the gut. A measure of splanchnic ischemia*.” Shortly thereafter, she became an associate professor at the University of Lund, and the main supervisor for seven PhD students there. Her research has focused largely on the abdominal wall including the open abdomen, and parastomal hernia in addition to the outcomes and quality of life after inguinal and incisional hernia repair. Agneta has also been involved in the development of several international guidelines from the European Hernia Society (EHS). Agneta has an h-index of 38 based on more than 100 original publications. Apart from the guidelines papers, some of her most cited papers were multicenter randomized controlled trials on recurrence rates after open and laparoscopic inguinal hernia repair and short-term outcomes after open and laparoscopic ventral hernia repair.

Agneta has been an active reviewer for many surgical journals and a member of the editorial boards of the British Journal of Surgery and the Scandinavian Journal of Surgery. In 2016, Agneta became an associate editor of the Hernia journal, and played an important role in raising the journal’s impact factor.

Agneta’s surgical career began with the introduction and development of the use of laparoscopy; Agneta was an active member and eventually became president of the Swedish Society of Laparoscopic Surgery. In 1996, Agneta became the head of the upper GI and the section of laparoscopy and abdominal wall reconstruction. With her colleagues, she developed the Malmö modified peritoneal flap repair for large incisional hernias and the local parastomal (LoPa) hernia repair, and their department became the premier centre in Sweden for handling complicated cases.

Agneta has been an active member of many surgical societies including the Council of the British Journal of Surgery and has also been the first female president of both the Swedish Surgical Society, the European Society for Surgical Research and the EHS. Furthermore, she has been a Board member of the Swedish Hernia Register and one of the founders of the Swedish Ventral Hernia Register. This underlines her strong interest in systematic quality control using surgical outcome registries.

**FIGURE 1:**
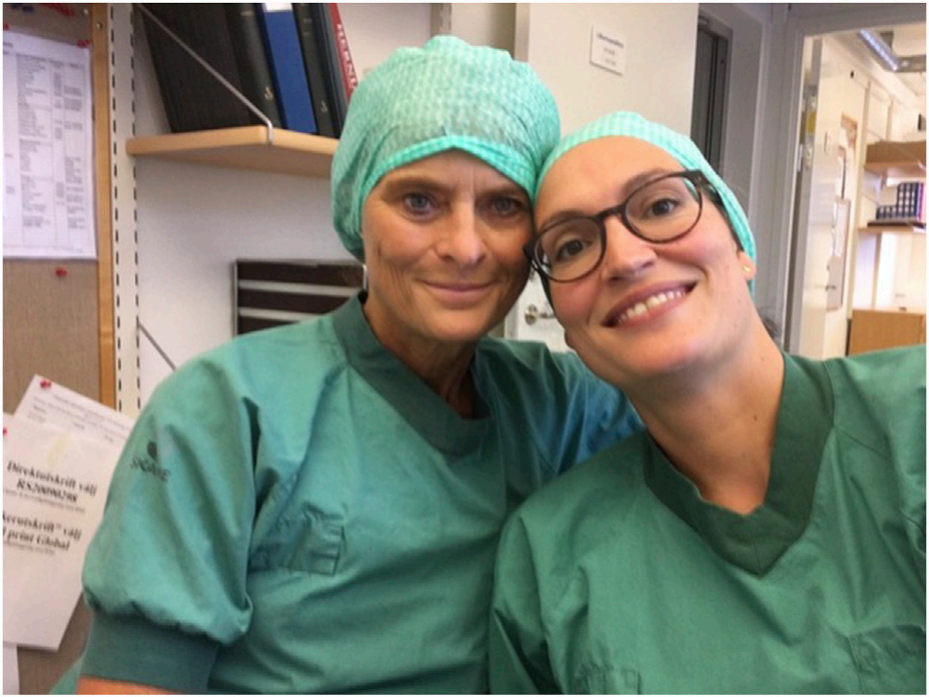
Picture of Agneta Montgomery and Nadia Henriksen at Malmø University Hospital, Sweden.

Agneta must have been used to being the only woman on many boards and being the first female president in some of them. Agneta never actively spoke about women’s rights, nor did she specifically promote women in surgery. However, Agneta became a member of these societies based on her merits and qualifications, and not because a woman was needed as a token. Agneta has always participated lively in discussions with a smile and by referring to evidence and facts. Agneta has been an ambitious supervisor in both surgery and research, expecting hard work and commitment from her students and co-workers alike (Figure 1). With a few of them, Agneta has to develop a structured education and training programme for surgeons in Sweden much of which remains applicable at an international level.

There is a natural respect around Agneta, and she consistently abstains from leveraging feminine allure as a strategy to attain her goals. Agneta has long been considered a respected and equally valued surgeon and scientist by the men around her. Agneta is indeed a role model for younger surgeons because of her former presence in different surgical societies and her great achievements in research and surgery. Agneta is an all-round loving and caring person and a very dear friend to many in hernia societies.

Agneta has retired from surgical practice and is currently supervising PhD students. She spends most of her spare time with her lovely husband, children, and grandchildren in Malmö or at her summer house in Mossby on the South coast of Sweden.

